# ASSOCIATION OF CARDIOVASCULAR DISEASE WITH RACE/ETHNICITY IN FAMILY CAREGIVERS OF PERSONS LIVING WITH DEMENTIA

**DOI:** 10.1093/geroni/igac059.2048

**Published:** 2022-12-20

**Authors:** Dawon Baik, Bryan McNair, Sophia Centi

**Affiliations:** University of Colorado, Aurora, Colorado, United States; Colorado School of Public Health, University of Colorado Anschutz Medical Campus, Aurora, Colorado, United States; CU Anschutz Medical Campus College of Nursing, Aurora, Colorado, United States

## Abstract

Family caregivers of persons with dementia (dementia FCGs) experience high caregiving burden, and caregiving-related stressors have been linked to cardiovascular disease (CVD). Racially and ethnically diverse FCGs face additional challenges in managing their own health; however, racial/ethnic disparities in caregiving-associated cardiovascular health are understudied and poorly understood. To fill the gap, we evaluated racial/ethnic differences in CVD risk and CVD conditions among dementia FCGs in the U.S. Using 2015-2020 Behavioral Risk Factor Surveillance System data (N = 6,132), we compared CVD risk and CVD conditions in racial/ethnic dementia FCG minorities to non-Hispanic White dementia FCGs. Logistic regression models were used to estimate unadjusted (OR) and adjusted odds ratios (AOR) with 95% confidence intervals (CI) for association of race/ethnicity with CVD risk and CVD conditions, adjusting for potential confounders. Compared to non-Hispanic White FCGs, non-Hispanic Black FCGs (AOR 0.33, 95% CI 0.2-0.53) and non-Hispanic Asian FCGs (AOR 0.16, 95% CI 0.05-0.51) were less likely to have depressive symptoms. Hispanic FCGs (OR 0.69, 95% CI 0.5-0.95), non-Hispanic Black FCGs (AOR 0.6, 95% CI 0.41-0.86), and non-Hispanic Asian FCGs (AOR 0.51, 95% CI 0.28-0.94) were less likely to smoke. Non-Hispanic Black FCGs were less likely to exercise (OR 0.69, 95% CI 0.52-0.91) and more likely to be obese (AOR 1.64, 95% CI 1.14-2.36). The findings confirm racial/ethnic disparities in psychological, behavioral, and metabolic risk factors for CVD among dementia FCGs. Future studies should investigate how dementia FCGs’ caregiving-related conditions affect CVD and how racial/ethnic considerations can inform culturally appropriate prevention-care strategies.

